# Pragmatism as a paradigm for patient‐oriented research

**DOI:** 10.1111/hex.13384

**Published:** 2021-11-08

**Authors:** Brooke Allemang, Kathleen Sitter, Gina Dimitropoulos

**Affiliations:** ^1^ Department of Social Work, Faculty of Social Work University of Calgary Calgary Alberta Canada; ^2^ Department of Psychiatry Mathison Centre for Mental Health Research & Education Calgary Alberta Canada

**Keywords:** paradigm, patient engagement, patient‐oriented research, pragmatic research, pragmatism, youth engagement

## Abstract

**Background:**

Mixed methods research studies continue to pervade the field of health care, where pragmatism as a research paradigm and patient‐oriented research (POR) as an engagement strategy are combined to strengthen the process and outcomes of the research. Pragmatists use the most appropriate research methods to address issues at hand, where complex social problems need multipronged approaches. As an emerging healthcare research strategy, POR actively engages individuals with lived experience across all stages of the research process. While POR continues to garner attention within mixed‐methods research designs, there is a paucity of literature that considers POR in relation to pragmatism.

**Objective:**

As POR grows in popularity within the field of health care, there is a need to explore the theoretical and epistemological alignment with pragmatism and the implications to research.

**Methods:**

To address this need, we provide a critical review of the literature to examine the synergies between POR and pragmatism, and argue for the adoption of pragmatism as a paradigm for conducting POR.

**Main Results:**

This article begins with a discussion of the philosophical underpinnings informing the pragmatic paradigm. It then identifies key alignments between POR and pragmatism across three intersecting concepts: democratic values, collaborative approaches to problem‐solving and the pursuit of social justice.

**Discussion and Conclusions:**

Reflecting on our experiences engaging with patient partners in a mixed‐methods POR study titled READY2Exit, we illustrate the relevance of pragmatism to POR by applying these concepts to practice. Implications and considerations for conducting POR within the pragmatic paradigm are also described.

**Patient or Public Contribution:**

This paper provides a critical review of the literature and did not directly involve patients or the public. The authors reflected on their experiences collaborating with five young adult patient partners in the READY2Exit study (case exemplar described in this article) to demonstrate the relevance of the pragmatic paradigm to POR. We acknowledge and thank the young adult patient partners for their contributions to the research, for encouraging us to think critically about patient engagement in research, and for sharing their experiences.

## 1 INTRODUCTION

A research paradigm is a theoretical framework comprised of a set of beliefs and values, which guides how research is conducted and knowledge conceptualized within scientific communities.^1^, ^2^, ^3^ Pragmatism as a paradigm is based upon the premise of utilizing the best methods to investigate real‐world problems, allowing for the use of multiple sources of data and knowledge to answer research questions.^4^, ^5^ This lends to its appropriateness for mixed methods research, whereby quantitative and qualitative data are collected and integrated within a single study, and multimethod research, which uses multiple forms of quantitative or qualitative data.^4^, ^5^, ^6^, ^7^, ^8^ Key tenets underlying the pragmatic paradigm include the utilisation of the scientific method of inquiry, whereby solutions are tested and either accepted or rejected, and alignment with democratic values.^9^ Pragmatists use the most appropriate research methods to address issues at hand, where complex social problems are in need of multipronged approaches. The adoption of the scientific method of inquiry, combined with values of democracy and social justice makes pragmatism a fitting paradigm for patient‐oriented researchers.

In this article, we describe the innate connection between patient‐oriented research (POR) and pragmatism drawing on the core concepts of democratic values, collaborative approaches to problem‐solving and the pursuit and maintenance of social justice. Indeed, as POR continues to grow in popularity within the field of health care, the theoretical and epistemological alignment with pragmatism further supports its adoption as the guiding paradigm when conducting POR.

This article begins with an overview of pragmatism and its theoretical roots. It proceeds with describing connections to POR while also drawing on a mixed‐methods case study exemplar to support this claim. The core concepts of democratic values, collaboration and social justice are further examined in connection to POR and pragmatism in the context of the healthcare field.

## 2 RESEARCH PARADIGMS

A research paradigm is a set of guiding values about scientific inquiry consisting of one's ontological commitments, epistemological beliefs and methodological preferences.^1^, ^2^, ^3^ The adoption of a paradigm supports the researcher in conceptualizing their beliefs about the nature of knowledge and selecting the methods best suited to address their research questions. Key features of four commonly used research paradigms (postpositivism, interpretivism, participatory, pragmatism) are summarized in Table 1 to provide a succinct overview of how these are situated in terms of ontology, epistemology, axiology and quality criteria. While individuals with lived experience are central to research, their level of involvement varies depending upon the paradigm and methods used. Much has been written about the roles of individuals with lived experience in transformative and participatory paradigms;^10^, ^11^ however, less is known about synergies between the pragmatic paradigm and participatory methods like POR. This article contributes to the literature by illustrating how pragmatism can be adopted to underpin POR studies.

**Table 1:** Summary of research paradigms, quality criteria and patient roles in research^a^

	Postpositivism	Interpretivism	Participatory	Pragmatism
Ontology	Critical realism—the reality is imperfectly apprehendable	Relativism—co‐constructed realities in local and specific contexts	Participative reality—subjective–objective reality	Reality is renegotiated and interpreted based on usefulness in specific contexts
Epistemology	Modified dualist/objectivist	Transactional/subjectivist; cocreated research findings	Extended epistemology of experimental, propositional and practical knowing; cocreated knowledge	Transactional realism; knowledge constructed based on interactions between people and their environments
Axiology	Reason, universal	Value‐laden, contextual	Value‐laden, transformative	Value‐laden, practical
Methodology	Modified experimental; falsification of hypothesis (including quantitative methods)	Hermeneutical/dialectical	Collaborative, action‐oriented inquiry; use of language grounded in shared experiential context	Mixed methods; action‐oriented inquiry; design‐based
Quality criteria	Internal/external validity, reliability, objectivity	Trustworthiness, credibility, dependability	Congruence of experiential, presentational and practical knowing; leads to transformative action in service of human flourishing	Provides a workable solution to problem, which prompted the research; leads to action/change
Patient roles in research^b^, ^c^	Learn/Inform, Participate (e.g., patients informed about a research project through social media channels, or patients enroled as study participants in a clinical trial)	Participate, consult (e.g., patients participate in a priority‐setting activity, or patients share lived experiences in a qualitative interview)	Collaborate, involve, lead/support (e.g., patients are lead investigators on a community‐based research project)	Consult, involve, collaborate, lead/support (e.g., patients sit on a standing advisory council for a clinical trial or patients involved as research partners)

^a^ Table adapted from Lincoln et al.^12^ and Heron and Reason.^13^

^b^ Patient roles/examples adapted from Vandall‐Walker.^14^

^c^ These roles are not prescriptive or mutually exclusive but are categorized here for the purposes of understanding how participants/patients are often engaged in research.

## 3 ROOTS OF CLASSICAL PRAGMATISM

The pragmatic paradigm arose out of the desire to focus efforts on solving practical problems in the real world through inquiry.^15^, ^16^ It relies heavily on the tenets of modern science, including the experimental method as a model for human problem‐solving.^15^ Pragmatism argues that experience is needed to ascribe meaning to an event.^17^ The goal, then, of pragmatic research is to utilize human experience as the primary means for building knowledge and understanding the world, as opposed to relying on absolute truths.^18^

Pragmatism provides an action‐oriented framework for research, wherein the researcher seeks to address practical issues arising directly from communities using the most appropriate methods for answering the research question.^19^, ^20^ John Dewey, one of the founding leaders of pragmatism, accepted a democratic and pluralistic way of life where social relations are characterized by cooperation, discussion, consultation and participation.^9^, ^18^ In pragmatism, it is argued that social problems are best defined by the individuals experiencing them, leading to the development of actionable research questions.^21^, ^22^ The inherent focus on social justice and joint problem‐solving in pragmatic research provide a strong rationale for the involvement of individuals with lived experience in the design and execution of research, one of the key pillars of POR. Congruence between the theoretical underpinnings of pragmatism and POR and implications for researchers will be explored in this article.

## 4 PRAGMATISM: THE INHERENT CONNECTION BETWEEN EXPERIENCE AND KNOWLEDGE

Epistemology, or one's theory of knowledge, is an important component of any philosophical stance.^23^ Pragmatism as a philosophy places an emphasis on action, presumed to be the most basic category of knowledge;^15^ more specifically on the consequences and meanings ascribed to actions.^17^ Dewey would argue that knowledge is constructed by interactions between humans and their environments, a concept he labels *transactional realism*.^15^ In pragmatism, inquiry using the scientific method allows for thoughtful decision‐making processes and choices aimed at reaching our intended outcomes.^24^, ^25^

Primary objectives of POR include conducting research that aligns with patient‐identified priorities and generating study findings that can be used to improve health systems.^26^ This is accomplished by engaging with individuals with lived experience of a health or mental health issue, informal caregivers, including family members friends and/or members of community organisations (henceforth referred to as ‘patient partners’) who bring the collective voice of specific, affected communities to research.^26^ Patient engagement occurs at various stages of the research through thoughtful dialogue and consultation to ensure methods and questions support their needs.^26^ The congruency between Dewey's conceptualisation of intelligent action and the deliberate involvement of patient partners in POR suggests that pragmatism is an appropriate philosophical stance to adopt when conducting this type of research.

According to the pragmatist, knowledge is explicitly linked with experience.^18^ Pragmatism recognizes the importance of the physical, psychological and social worlds, including culture, language, institutions and subjective thoughts.^20^ Knowledge is ‘both constructed and based on the reality of the world we experience and live in’,^20^ implying that although knowledge does exist in the external world, it must be *experienced* by individuals. Herein lies another connection between pragmatism and POR; the value placed on *experiential knowledge*. POR's privileging of the patient voice by seeking active involvement of those with lived experience^26^ suggests an inherent alignment with pragmatism's conceptualisation of knowledge.

Our experiences and actions take place within particular historical, social and cultural contexts, therefore, there may be limits on our ability to use previous experiences to predict the outcomes of future actions.^24^ Inquiry is needed because we cannot always rely on our past experiences, given our assumptions are viewed as fallible.^24^ The process of inquiry involves careful, conscious and reflective decision‐making before action using the scientific method.^27^, ^28^ We develop warranted assumptions by taking certain actions and experiencing the resulting outcomes.^27^ The outcomes gleaned from the process of scientific inquiry then guide our future behaviours and actions. In the field of POR, results are often applied directly in clinical practice or policy development^26^ given the collaborative nature of the research process, explicating another connection between pragmatism and POR.

Pragmatism does not privilege one type of knowledge or research method over another. Instead, it calls on researchers to critically analyse ‘which interests are served in a particular situation by the application of a particular kind of knowledge’.^29^ This pluralist approach allows for the acceptance and recognition of validity amongst different perspectives, interests and forms of knowledge.^29^ Because pragmatism is accepting of different ways of knowing, it does not attempt to position particular forms of evidence in a hierarchical structure.^19^ This allows for careful consideration of what type of knowledge would best serve the interests of a community within a given context, including the value of experiential knowledge contributed by individuals with lived experience.

Pragmatism asserts that the paradigm wars, concerning false dichotomies between quantitative and qualitative methods, are over, as this polarisation does not promote the advancement of the health science field.^30^ Thus, this paradigm supports the adoption of different methods of inquiry to address problems in the most appropriate way, recognizing that methodologies are tools used to aid in understanding the world.^30^ Indeed, pragmatism allows for the use and mixing of different data sources based on the premise that their consequences are developed in the processes of the ongoing inquiry.^22^, ^28^ It, therefore, serves as a paradigm accepting of mixed and multimethod research. While multiple paradigms may be used when conducting mixed methods research,^31^, ^32^, ^33^, ^34^, ^35^, ^36^, ^37^ this paper argues that pragmatism's roots in social justice and democracy, combined with the resolve to produce tangible outcomes make it a fitting worldview to adopt when conducting mixed or multimethod studies in the healthcare field. There are clear connections between the principles of pragmatism and POR, which will be explored in the next section.

## 5 THE RELEVANCE OF PRAGMATISM IN POR

POR involves actively engaging with individuals from the population of interest in various aspects of health research from conceptualisation and design to governance, execution and dissemination of findings into clinical practice.^26^, ^38^ It finds its roots in well‐established collaborative research approaches including participatory action research and patient/public involvement in research.^39^, ^40^ POR is founded on the principles of mutual respect, support and coconstruction of the research, and places inherent value on the patient as an expert.^26^

Within a POR framework, patient partners are critical in identifying research priorities, defining questions and collaborating with researchers to develop appropriate methods for answering these questions.^26^ POR seeks to move the patient role in research from ‘subject’ to ‘expert’, supporting a shift from the previously dominant paradigm in which power imbalances between researchers and patients prevailed.^41^ The concept of patient engagement in POR has been heavily influenced by the field of participatory action research, calling for collaboration between community members and researchers across all phases of research.^40^, ^42^, ^43^ According to Vandall‐Walker's^14^ continuum of patient engagement in POR, patient roles in the research process vary depending on the research question, resources available (e.g., time, funding), goals of the project and patient preferences. Patient engagement in research ranges from learning (e.g., patients informed about the project through social media channels) and participating (e.g., patients take part in a study by completing a survey), to consulting (e.g., patients involved in a priority‐setting activity), being involved (e.g., patients sit on standing advisory council), being collaborators (e.g., patients involved as research partners) and to leading research (e.g., community‐based research projects where patients are lead investigators), with patients playing more prominent roles the further along the continuum the study is positioned.^14^ These six patients' roles^14^ are used in Table 1 to highlight how the responsibilities of patients vary based on the paradigm informing the research.

Leveraging the complementary strengths of researchers and patient partners produces powerful research teams, which are well‐positioned to address real world, pressing issues in health care.^44^ The perspectives of both groups allow for a multi‐faceted approach to research, where diverse insights and voices are valued and results are responsive to the needs of patients.^26^ In fact, pragmatic clinical trials have become commonplace in the POR landscape, with patient partners providing their perspectives on enhancing the practicality of clinical trials, and ensuring the methods for data collection are responsive to the needs of participants.^45^ For instance, measuring patient‐reported outcomes, which capture patients' perceptions of their quality of life or symptoms, as opposed to clinician‐administered tools, ‘may enhance the real world nature of data collection within the clinical trial’.^45^ Emerging evidence suggests that outcomes like improved participant enrolment, decreased attrition and enhanced dissemination of study findings have been achieved using POR approaches.^39^ Further, some of the challenges that may arise when conducting POR, including power imbalances and conflicting ideas about priorities for research, can begin to be addressed by adopting a pragmatic approach. Engaging with patient partners from the outset of project conception, clearly defining patient/researcher roles and responsibilities, and using collaborative priority setting activities, like James Lind Alliance Priority Setting Partnerships,^46^ are examples of how pragmatism can serve to enhance the practicality of POR studies.

Pragmatism's emphasis on context, time and place, requires that knowledge generated from research be considered within these confines.^24^ Applications of proposed solutions to specific local contexts may be supported by the inclusion of patient partners in research. Given pragmatism and POR both strive for social change that is meaningful to local stakeholders, the pragmatic paradigm is put forth as a fitting approach for POR. To illustrate the relevance of pragmatism to POR, we will discuss how the concepts of democratic values, collaborative problem‐solving and social justice apply to a mixed methods POR study titled READY2Exit. We acknowledge, however, that POR projects may adopt a wide range of methods, including quantitative, qualitative, mixed or multiple methods depending upon the research question and study objectives.

## 6 POR CASE EXAMPLE: THE READY2EXIT STUDY

This case exemplar draws on the first author's research; a 3‐year mixed‐methods POR project focused on the readiness and experiences of adolescents with co‐occurring health and mental health issues exiting paediatric services (READY2Exit) in Alberta, Canada.^47^ The study is a doctoral research project, which aims to expand our understanding of the needs and experiences of young adults with both physical and mental health issues as they transition from paediatric to adult services. It is well known that the paediatric‐adult transition for adolescents with chronic health conditions is a high‐risk period^48^ and that assessing transition readiness is a core component of best practice transition planning.^49^ ‘Transition readiness’ refers to the extent of an adolescent's health‐related knowledge (e.g., ability to describe condition) and self‐management skills (e.g., filling prescriptions independently, making appointments) before transfer.^50^ Though a large proportion (up to 57%) of individuals with chronic health conditions also experience mental health issues (e.g., depression, anxiety),^51^, ^52^ transition readiness for adolescents with *co‐occurring* health and mental health issues has not been adequately explored. The objectives of READY2Exit are to (1) describe the association between transition readiness and mental health comorbidities in adolescents with chronic health conditions, and (2) to better understand the experiences of this group as they prepare to exit paediatric services.^47^ These goals are being addressed using a patient‐oriented, mixed‐methods approach. READY2Exit is currently in the data collection and analysis stage. Presently, we are collecting and analyzing quantitative data corresponding to objective #1, and are codesigning the qualitative interview guide and recruitment materials to address objective #2. Qualitative interviews are scheduled to take place within the next 3–4 months.

### 6.1 READY2Exit young adult engagement strategy

READY2Exit is being conducted in collaboration with a project‐specific Young Adult Advisory Council (YAAC).^47^ The READY2Exit YAAC was established in March 2021, consisting of five female‐identified young adults (aged 18–30) with lived/living experience in the health and/or mental health systems in Canada. Before the development of the YAAC, a series of consultations were held by the doctoral student with existing advisory councils (i.e., Child and Youth Advisory Council at Alberta Children's Hospital) to identify priorities for this project, refine the overarching research questions and adjust the language used throughout the proposal. Specifically, brainstorming activities about the most challenging aspects of health care transition were carried out, where readiness for transition, the emergence of mental health concerns and self‐management emerged as priorities from the councils. These priorities were used to develop the READY2Exit study objectives, and the councils assisted in refining the research questions (i.e., adjusting language) and identifying methods (i.e., the importance of qualitative interviews).

YAAC members came to the project with varying levels of experience being involved in advisory council work, and knowledge surrounding health/mental health research. While young adult engagement activities were initially planned to take place at quarterly, in‐person meetings at the study's academic institution, the COVID‐19 pandemic necessitated a shift to virtual platforms for engagement. This allowed for the recruitment of YAAC members from a geographically diverse area, so long as they had internet access and their own device. The shift to virtual meetings called for attention to how rapport would be developed between members online, group dynamics and preparation of materials/activities suited to an online platform. A clear terms of reference document was critical to ensuring the YAAC members felt safe contributing to conversations by Zoom and allowed for transparency in codesigning group norms and guidelines for online engagement. Specifically, the doctoral student conducted an environmental scan to identify terms of reference documents being used by well‐established youth advisory councils. A draft terms of reference document was prepared by the doctoral student, with headings including guiding principles, confidentiality, engagement options and roles and was circulated amongst the group. YAAC members then provided their edits on the document via email. Suggested changes included the incorporation of a land acknowledgement. The terms of reference were then discussed at a group YAAC meeting, where additional feedback was obtained verbally, and the final document received approval from all members.

Our first READY2Exit YAAC meeting took place over Zoom in April 2021, where we completed icebreakers and introductions, agreed upon our terms of reference, reviewed the study and discussed everyone's hopes for collaborating. We determined that we would continue to meet monthly for an hour and a half over Zoom. At our second meeting in May 2021, we decided to create a shared drive folder where meeting slides, minutes, educational resources, training opportunities, terms of reference and study materials would be stored. The YAAC identified they could then review materials on their own time, with this option being preferable to receiving documents via email. We also reviewed a widely used transition readiness assessment tool (Transition Readiness Assessment Questionnaire^53^), with YAAC members providing their input on survey items, and identifying areas for exploration in the qualitative phase of READY2Exit.

While we acknowledge the value of engaging patient partners throughout all study phases, we recognize there are certain milestones to achieve within the confines of a time‐limited doctoral study. Thus, we opted to utilize the Involvement Matrix, a tool codesigned by patients, carers and researchers, within the READY2Exit YAAC, allowing members to decide which role(s) they would like to adopt (i.e., Listener, Co‐thinker, Advisor, Partner, Decision‐maker) in which phase(s) of the research (i.e., Preparation, Execution, Implementation).^54^ We reviewed the Involvement Matrix^54^ together and brainstormed a list of potential tasks associated with READY2Exit (e.g., codevelopment of the interview guide, recruitment, member checking, knowledge translation, social media outreach). Each YAAC member was invited to reflect on their own skills, interests and goals and to complete the Matrix^54^ accordingly, by identifying how they wanted to contribute to each task. This allowed for a structured and transparent discussion about tasks, responsibilities and timelines, while offering the members a sense of choice and ownership over their preferred roles within READY2Exit.

As a group, we determined we would alternate monthly meeting chairs to support YAAC members in gaining experience with group facilitation. YAAC members were also encouraged to reflect upon their strengths, and which skills they would like to develop while involved in this council (e.g., public speaking, networking, research). Based on YAAC members' suggestions, we are collectively designing a peer mentorship structure, where members who are strong public speakers will provide support to those who would like to improve on this skill, for instance.

As described in this case exemplar, actions were taken in developing READY2Exit and the YAAC that are reflective of a pragmatic approach to research. Specifically, the use of the Involvement Matrix and the flexibility required to shift to virtual engagement during the pandemic align with underlying principles of pragmatism, including a focus on what works, being flexible and collaborating with key stakeholders. In the following section, three specific concepts will be described, which further connect pragmatism with the values of POR.

## 7 CONGRUENCE BETWEEN PRAGMATISM AND POR: THREE OVERLAPPING CONCEPTS

Congruence between pragmatism and POR is evident in the following three concepts: democratic values, collaborative approaches to problem‐solving and the pursuit of social justice. With reference to the case exemplar, these synergies are described to clearly illustrate these concepts in practice.

### 7.1 Democratic values

Underlying pragmatic research is Dewey's guiding principle of democracy, where the goal is to minimize imbalances of power by engaging communities with differing perspectives to obtain their input on priorities for inquiry.^18^ The centrality of democratic values within pragmatism supports the use of pragmatism when conducting POR. A shared responsibility for problem‐solving, flexible negotiation and open dialogue to come to mutually desirable research questions are principles aligning with both the democratic model within a pragmatic research paradigm and POR. As evidenced by our case example, YAAC members are bringing their unique voices to the research, making decisions about how they would like to be involved in the research, conduct meetings and develop mentorship and learning opportunities. The codesigned terms of reference and meeting structure provide YAAC members an opportunity to actively contribute to problem‐solving within READY2Exit and to come to flexible, and mutually agreed‐upon goals.

Democratic values underlying the pragmatic paradigm and POR include collaboration, citizen engagement, promoting a sense of community, relationship‐building and a focus on serving the public interest.^26^, ^55^, ^56^ Pragmatism has been utilized as a guiding paradigm in action research given its attempts to address real‐world problems identified by those with lived experience through democratic inquiry.^10^ Democratic inquiries privilege certain epistemic practices related to problem‐solving (i.e., empirical methods), communication (i.e., open dialogue, active listening) and attitudes (i.e., innovative, creative, future‐oriented) over others.^18^ In fact, patient engagement in POR is viewed as vital to generating meaningful, relevant research, which aligns with democratic values.^57^, ^58^ Examples of democratic epistemic practices in READY2Exit include the use of open‐ended questions during group brainstorming, creative rapport‐building activities to begin and end each meeting, and future‐oriented discussions about the use of digital media for knowledge translation. These represent clear connections between POR and pragmatism surrounding democratic values within READY2Exit.

### 7.2 Collaborative approaches to problem‐solving

According to Dewey,^28^ ‘any problem of scientific inquiry that does not grow out of actual (or “practical”) social conditions is factitious; it is arbitrarily set by the inquirer’. The process of working collaboratively with community members ‘characterizes both the pragmatic method of inquiry and the democratic model’.^9^ A shared responsibility for problem‐solving, flexible negotiation and open dialogue to come to mutually desirable research questions are guiding principles within pragmatism and POR.^26^

Motivations for health researchers to collaborate with patient partners in research vary. Goals for engaging patient partners in research may be transactional in nature, reflecting efforts to achieve certain outcomes like increased relevance of the research to key stakeholders or higher retention rates in clinical trials.^57^ Other motivations may relate to the shift away from paternalism in health care and towards a more inclusive and accountable research agenda.^38^, ^41^, ^57^ A moral stance to POR dictates an imperative to collaborate with patient partners in designing research that will inform the policies and programmes serving them.^58^, ^59^ This joint approach to research gives rise to important processes for researchers, including an obligation to reflect on one's underlying values, biases and motivations for conducting the research.^60^ Engagement with YAAC members who have different backgrounds, abilities, social locations and experiences within health and mental health systems supports a pragmatic approach to research, wherein we collectively reflect on our positionality and discuss how we approach research and determine outcomes. This has afforded us opportunities to determine how individual, familial and systemic factors contribute to our assumptions, values and priorities within the research. YAAC members' motivations for becoming involved in READY2Exit included a desire to give back and improve care transitions for other youth, a passion for the subject area given their own experiences navigating life transitions, hopes for connecting with peers and an interest in developing new skills/capacities (e.g., research, leadership, teamwork). In YAAC meetings, the first author adopts an open and curious stance, characterized by asking open‐ended questions, which allows members to steer conversations and express their views, and actively listens to capture the priorities of YAAC members. This supports a collaborative approach to problem‐solving, ensuring READY2Exit is exploring areas of concern for the YAAC.

### 7.3 Pursuit of social justice

An important principle in pragmatic research is the commitment to producing a positive change in the world by addressing a problem through experimentation in context. Hall^22^ asserts that any knowledge gained through pragmatic research should be shared with communities, clinicians and key stakeholders to improve society and address the conditions that prompted the research in the first place. The pragmatic paradigm operates in pursuit of social justice using inquiry that is action‐oriented and context‐specific.^10^ Pragmatism and POR are connected in that both aim to generate knowledge through inquiry that warrants *action* and stimulates *change*. Connections between POR and pragmatism centred around improving societal conditions further illustrate the relevance of pragmatism as a guiding paradigm for POR.

A strength of POR is that both researcher and patient partner bring their unique opinions, experiences, social locations and knowledge bases into mutually beneficial working relationships to delve into social problems collaboratively.^10^, ^26^, ^44^ Researchers and patient partners work together to define research problems, gather and analyse relevant data with the goal of improving policies, programmes and practices.^10^, ^61^ Researchers operating within the POR approach bring knowledge of specific research methods, whereas patient partners have critical ‘insider’ knowledge of social problems from their lived experiences and connections with other ‘insiders’ who may be able to provide additional insights.^10^ As a result, strong partnerships are formed between researchers and patient partners, positioning them well to address real world, pressing issues within health care.^44^

During initial meetings of the YAAC, the first author delivered interactive presentations on concepts like POR, mixed methods research and knowledge translation to support council members in developing their research skills. In addition to compensation, educational resources, presentations and question and answer periods on specific research methods of interest to YAAC members allow for reciprocal learning, growth and mentorship. To date, YAAC members have offered critical insights on their experiences completing transition readiness assessments and communicating with health care providers in preparation for service transitions. Their perspectives have allowed for a more nuanced understanding of transition planning, and how this differs based on factors, such as diagnosis, care provider, geographic location and parental/caregiver involvement. Our rich conversations offer a space for reflection on how issues of access, systemic oppression, racism, socioeconomic status, health literacy and capacity factor into transition readiness and service navigation. All of these discussions support the development of a social justice‐oriented lens to the research in which we look beyond a single transition readiness score and consider how individual, familial, community and macrolevel factors shape transition experiences. These ideas are supported by the adoption of the social–ecological model of adolescent and young adult readiness to transition (SMART) within the research, which acknowledges how parent, provider and systemic factors influence the acquisition of self‐management skills in youth with chronic conditions.^62^

## 8 METHODOLOGICAL CONGRUENCE BETWEEN READY2EXIT AND THE PRAGMATIC PARADIGM

Pragmatism guided the selection of READY2Exit's methods, theoretical framework and patient engagement strategy. We elected a mixed‐methods, POR approach informed by the SMART model,^62^ as this allowed for a comprehensive and multi‐faceted outlook on transition readiness that would serve to enhance the applicability of our findings to clinical practice. To highlight the methodological congruence between READY2Exit with the pragmatic paradigm, we have illustrated how multiple sources of data (e.g., questionnaires, qualitative interviews and patient partners' lived experiences) can be combined to best address the research questions, and increase the practical uptake of the results by clinicians, youth and families. Our patient engagement plan evolved based on environmental factors (i.e., COVID‐19, funding to support patient partner compensation), project timelines (i.e., time‐limited doctoral programme) and the preferences of YAAC members regarding their desired level of involvement. This flexibility is a common feature of pragmatic research, where adjustments are made to enhance the real‐world utility of the methods and results.^45^ The integration of quantitative and qualitative data on transition readiness, while working in partnership with YAAC members to problem solve and adapt our study materials to best reach youth, demonstrates how a mixed‐methods, POR project operates within the pragmatic paradigm.

By approaching POR with an intentional research paradigm guiding the work, researchers will be armed with the knowledge and tools to carry out studies that are internally coherent in terms of epistemology, axiology and ontology. As we have demonstrated, we advocate for the adoption of the pragmatic paradigm in the field of POR given the goals of addressing problems arising within communities and producing societal change that is meaningful to local stakeholders are common to both pragmatism and POR.

## 9 CONCLUSION

Health researchers are required to consider the alignment of paradigm, epistemological stance, methodology and methods in designing coherent and meaningful studies. Throughout this paper, we have made the case that the pragmatic paradigm is well‐suited for POR, given the alignment of their underlying principles. We identified parallels between the concepts of democratic values, collaborative approaches to problem‐solving and the pursuit of social justice in pragmatism and POR, and used a case example to demonstrate their application in practice. This paper provides a critical contribution to the literature by examining the epistemological and theoretical underpinnings of pragmatism, and synergies with public participation in health research. It seeks to further develop the growing field of POR by arguing for thoughtful consideration of research design, reflection upon the core values of this study and its alignment with the pragmatic paradigm.

Pragmatism's focus on addressing real‐world problems, as defined by the communities within which these occur, makes it a natural guiding approach for health researchers who value collaborations with patient partners. Using the READY2Exit case example, we have illustrated that the adoption of the pragmatic paradigm, wherein the emphasis is on what works within a particular time, place and local context, lends itself well to POR approaches. Health researchers wanting to incorporate the principles of pragmatism into POR should adopt a flexible approach given the iterative nature of pragmatic, patient‐engaged research, consider the resources available to them (e.g., time, funding) and their positionality in relation to the research (i.e., values, epistemological beliefs, ontological commitments).

We have also argued that health researchers operating within the pragmatic paradigm are well‐positioned to conduct POR, given its action‐oriented, democratic approach to inquiry. A value‐based approach to POR, where the inclusion of patient partners is viewed as a moral imperative, can allow researchers to conduct studies aligned with democratic values, while honouring the patient voice and advocating for change. Considering the epistemological and ontological assumptions of pragmatism and how these apply to the growing field of POR is a frontier that requires further exploration.

## CONFLICT OF INTERESTS

The authors declare that there are no conflict of interests.

## Data Availability

Data sharing is not applicable to this article as no datasets were generated or analysed during the current study.
